# Multifocal Extrapulmonary Tuberculosis Presenting with a Masticatory Space Abscess

**DOI:** 10.1590/0037-8682-0438-2025

**Published:** 2026-02-06

**Authors:** Ragıp Afşın Alay, Elif Gözgeç, Alperen Aksakal, Handan Alay

**Affiliations:** 1 Department of Child Health and Diseases, Faculty of Medicine, Ataturk University, Erzurum, Turkey.; 2 Department of Radiology, Faculty of Medicine, Ataturk University, Erzurum, Turkey.; 3 Department of Pulmonary Diseases, Faculty of Medicine, Ataturk University, Erzurum, Turkey.; 4 Department of Infection Diseases and Clinical Microbiology, Faculty of Medicine, Ataturk University, Erzurum, Turkey.

A 14-year-old girl with autism presented with persistent right cervical swelling for eight months. She had undergone multiple abscess drainages and prolonged antibiotic therapy without improvement. Cultures and serologic tests for Francisella tularensis, EBV, CMV, and Brucella were negative. Tuberculosis testing (purified protein derivative [PPD] 9 mm, QuantiFERON negative, gastric aspirate smear, and polymerase chain reaction [PCR] negative) was initially inconclusive. An excisional lymph node biopsy revealed no caseating necrosis and was negative for acid-fast bacilli. Family history revealed maternal pulmonary tuberculosis eight years earlier, without household prophylaxis.

On admission, she reported night sweats, a 5-kg weight loss, and persistent lymphadenopathy. Physical examination showed cervical lymph nodes with a chronic draining fistula (Figure 1). Chest computed tomography (CT) and neck magnetic resonance imaging (MRI) demonstrated necrotic mediastinal lymphadenopathy, parenchymal consolidations, paravertebral rib destruction, and a masticatory space abscess (Figures 2 and 3). Endobronchial ultrasound-guided bronchoalveolar lavage culture yielded Mycobacterium tuberculosis. The patient was treated with rifampicin, pyrazinamide, streptomycin, and linezolid due to isoniazid resistance, resulting in clinical improvement.

**FİGURE 1:**
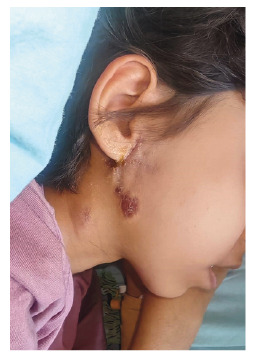
External draining fistula and scar in the right auricular-submandibular region.

**FİGURE 2:**
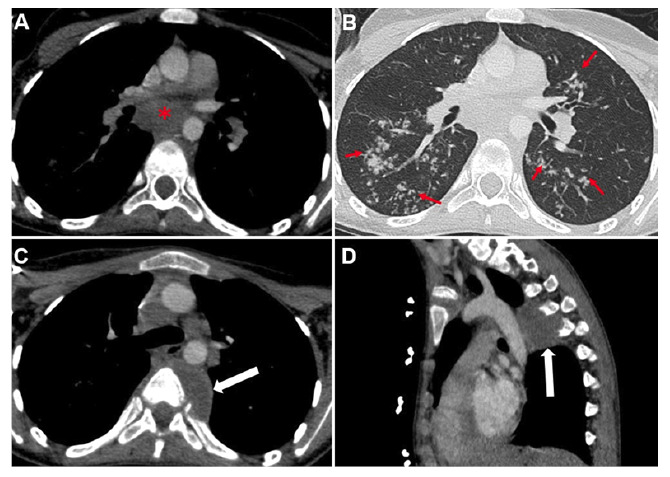
Axial non-contrast chest CT, soft tissue window **(A)**, demonstrates necrotic lymphadenopathy (asterisks) in the mediastinum and bud-like branch patterns, as well as patchy consolidated areas (arrows) in the bilateral parenchyma on the parenchymal window **(B)**. Axial **(C)** and sagittal **(D)** non-contrast chest CT images show a hypodense lesion causing rib destruction in the left paravertebral region (arrow).

**FİGURE 3:**
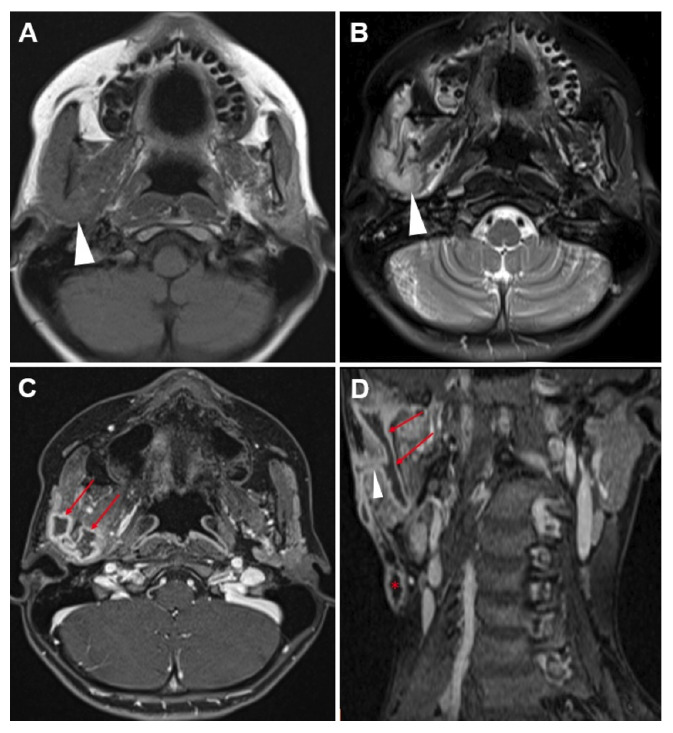
Axial MR images show a hypointense appearance on the T1-weighted image **(A)** and a hyperintense appearance (arrow) on the T2-weighted image **(B)** in the right masticatory space. Post-contrast T1-weighted axial **(C)** and coronal **(D)** sections demonstrate a peripherally contrast-enhancing abscess loculation (red arrows) and a fistula tract (arrowhead) extending to the skin. A central necrotic LAP formation (asterisks) is noted inferior to the lesion.

Extrapulmonary tuberculosis remains challenging to diagnose because clinical samples obtained from relatively inaccessible sites are often paucibacillary, reducing the sensitivity of conventional diagnostic tests^1^. Persistent cervical lymphadenitis with a draining fistula should prompt repeat tuberculosis evaluation, even when initial PPD, QuantiFERON, culture, and PCR results are negative^2^. Imaging (MRI/CT) is crucial to delineate abscess tracts, lymph node necrosis, and bone involvement, thereby guiding both diagnosis and management^3^.

## References

[1] Lee JY (2015). Diagnosis and treatment of extrapulmonary tuberculosis. Tuberc Respir Dis.

[2] Fontanilla JM, Barnes A, Fordham von Reyn C (2011). Current diagnosis and management of peripheral tuberculous lymphadenitis. Clin Infect Dis.

[3] Pattamapaspong N, Kanthawang T, Peh WCG, Hammami N, Bouaziz MC, Ladeb MF (2024). Imaging of thoracic tuberculosis: pulmonary and extrapulmonary. BJR Open.

